# Metagenomics data of microbial communities of natural organic matter from the dispersion train of sulfide tailings

**DOI:** 10.1016/j.dib.2021.106720

**Published:** 2021-01-08

**Authors:** Anton V. Korzhuk, Irina N. Myagkaya, Alexei S. Rozanov, Nikita I. Ershov, Bagai-ool Yu. Saryg-ool, Victor I. Malov, Maria A. Gustaytis, Aleksandra A. Shipova, Elena V. Lazareva, Sergey E. Peltek

**Affiliations:** a Laboratory of molecular biotechnology, Institute of Cytology and Genetics, Siberian Branch of the Russian Academy of Sciences (ICG SB RAS), 10 Lavrentjeva Ave., Novosibirsk 630090, Russia; b Kurchatov Genomics Center, Institute of Cytology and Genetics, SB RAS, 10 Lavrentjeva Ave., Novosibirsk 630090, Russia; c V.S. Sobolev Institute of Geology and Mineralogy, Siberian Branch of the Russian Academy of Sciences (IGM SB RAS), Novosibirsk 630090, Russia

**Keywords:** Metagenomics, 16S rRNA, Sulfide tailings, Natural organic matter metagenome

## Abstract

Below is data on the microbial diversity of natural organic matter from the Dispersion Train of Sulfide Tailings (northern Salaire Ridge, southwestern Siberia, Russia, Ursk Village). Data was obtained using 16s rRNA amplicon directed metagenomic sequencing on Illumina MiSeq. The raw sequence data used for analysis is available in NCBI under the Sequence Read Archive (SRA) with BioProject No. PRJNA670045 and SRA accession number SRX9314152, SRX9314376. The data sequences of the 16s rRNA gene are presented at the links MW142408-MW142413, MW142414-MW142447.

## Specifications Table

**Table:** 

Subject	Environmental Genomics and Metagenomics
Specific subject area	High sulfide mine tailings metagenomics
Type of data	Figures and 16S rRNA amplicon sequencing data
How data were acquired	Illumina MiSeq platform, QIIME2 v.2020.2
Data format	Raw and analyzed
Parameters for data collection	Metagenomic RNA isolated from NOM samples were prepared by amplifying the V3-V4 region of the 16S rRNA gene paired-end sequenced on an Illumina MiSeq platform.
Description of data collection	Metagenomic DNA extraction, amplicon sequencing of V3-V4 region of 16S rRNA gene and paired reads processing using QIIME2 v.2020.2 platform
Data source location	Ursk tailings, Kemerovo region, Russia: 54.446756N, 85.403567E
Data accessibility	Raw sequencing data: Repository name: NCBI SRA Data identification number: SRX9314152 (Sample 1) and SRX9314376 (Sample 2) Direct URL to data: https://www.ncbi.nlm.nih.gov/sra/SRX9314152 (Sample 1) and https://www.ncbi.nlm.nih.gov/sra/SRX9314376 (Sample 2)

## Value of the Data

• The data provide complete taxonomic profiles of microbial diversity and abundance in highly contaminated acidic environment with high contents of sulfates, and can also provide an initial picture of the functionality of lithotrophs inhabiting the environment;
• The data will be of interest to researchers studying the phylogeography of extremophile prokaryotes and/or searching for attractive organisms/genes for industrial use;
• In the future, the data can be used for profiling, annotation, or reconstruction of pathways for understanding the metabolic processes of the microbial community that thrives in lithotrophic conditions and searching for genes responsible for the acceptance and concentration of rare earth metals, precious metals (gold and silver), nonferrous metals (zinc) as well as selenium, iodine and mercury.

## 1 Data Description

**Fig. 1:**
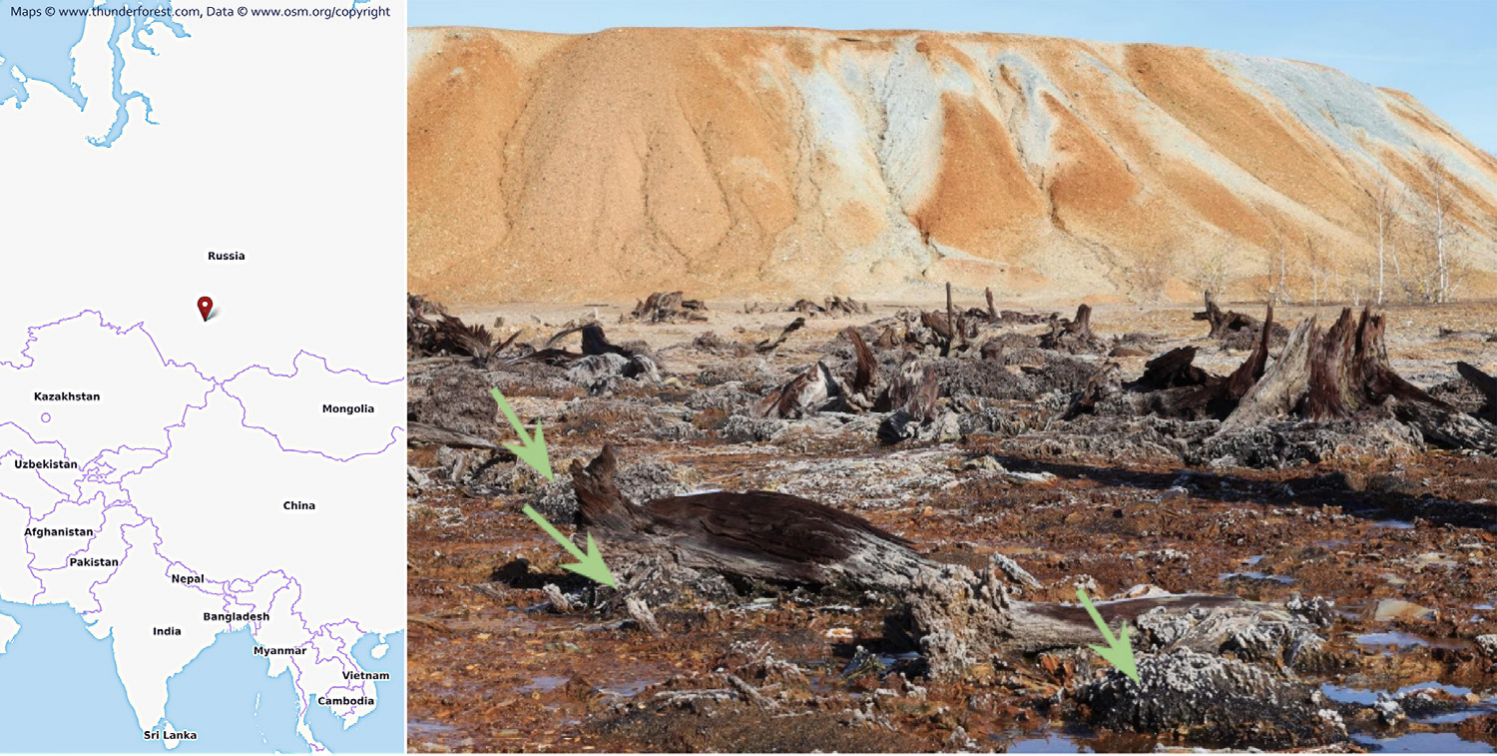
Sampling location. NOMs are marked with arrows in the foreground. The dump of wastes II is in the background.

There are Ursk tailings near the residential village of Ursk (Kemerovo region, Russia). They're composed of cyanidation wastes of primary high-sulfide polymetallic ores (wastes I) and wastes of oxidation zone ores (wastes II) of the Novo-Ursk deposit. For more than 80 years such unfixed wastes had been carried to the underlying boggy ravine, where there had been a long-term interaction of natural organic matter (NOM) with wastes and acid mine drainage (AMD) formed due to oxidative leaching of wastes. Here, authigenic minerals formation (sulfates; secondary aluminosilicates; Fe sulfides: framboidal pyrite; Zn sulfides; Hg sulfides; Hg selenides; Ag iodides; Au^0^) had previously been established in NOM, which is associated with the concentration of the corresponding elements: 11700 ppm Hg, 41,300 ppm Zn, 6060 ppm Se, 155 Au ppm, 534 Ag ppm, 416 ppm I. These processes were mainly observed in part of dispersion trains covered with wastes II [1]. There are the remnants of sedge and trees undecomposed due to the influence of AMD and wastes. As a result, ecological niches have been formed, in which there is a substance with a pronounced lithotrophic origin, which interacts with the organic surface material. NOM interacting with wastes II was sampled in June 2019 (Fig. 1, Table 1).

**Table 1:** Description of the sampling points.

Name	рН	Description
Sample 1	2.7	NOM buried under wastes II. Coordinates: 54.446756N, 85.403567E
Sample 2	2.7	NOM with cemented, hard crusts of Fe hydroxides. Coordinates: 54.446756N, 85.403567E

Raw sequencing data of Sample 1 contain 36.820 paired-end reads with a length of 301 bp totaling 22.2M base pairs. As for Sample 2, 44.808 paired-end sequences with a length of 301 bp totaling 27M base pairs were obtained.

In total, after processing and cleaning, 11 OTUs including 181 sequences and 38 OTUs including 6309 sequences were obtained from Samples 1 and 2 respectively.

**Fig. 2:**
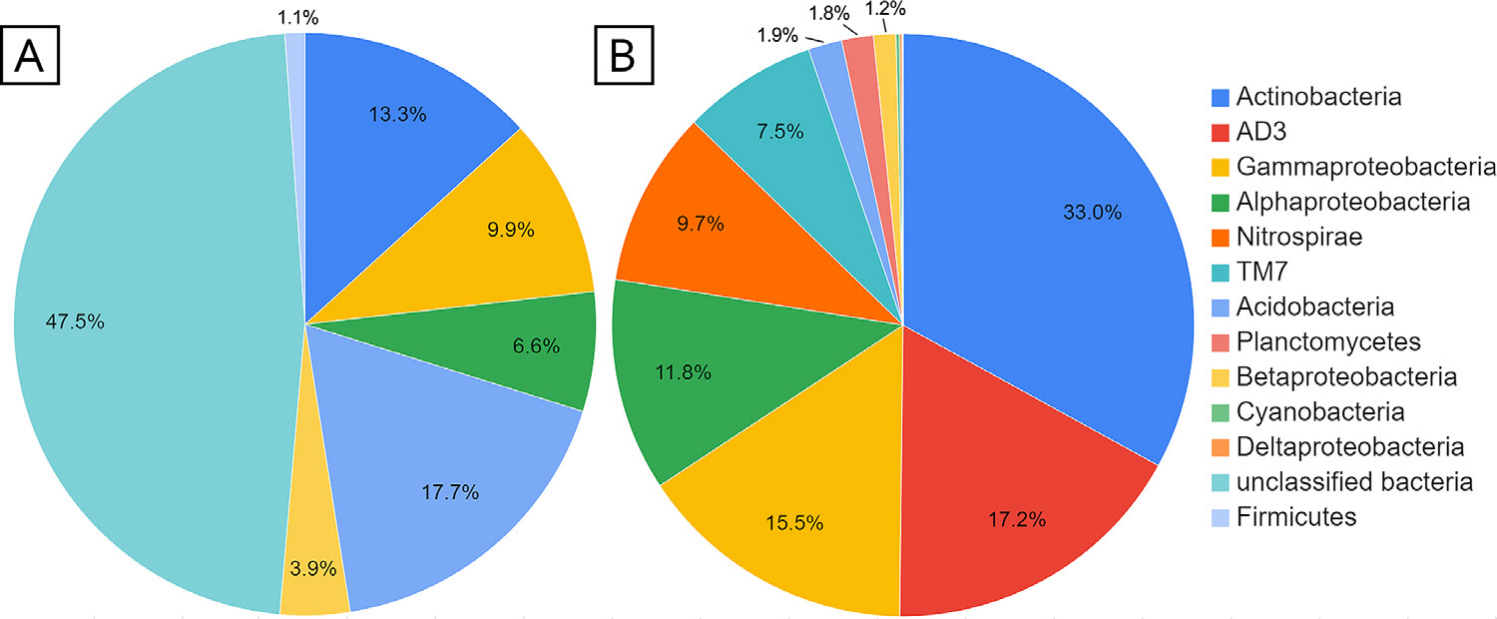
Charts of the taxonomic composition of communities: A - Sample 1; B - Sample 2.

In the microbial community of Sample 2 (Fig. 2), bacteria predominated: 33% were representatives of Actinobacteria (Acidimicrobiales; Actinomycetales; Acidobacteriales;), 17.2% were representatives of the candidate branch AD3 (JG37-AG-4); 15.5% Gammaproteobacteria (Xanthomonadales; Pseudomonadales;), 11.8% Alphaproteobacteria (Rhodospirillales, Rhizobiales), 9.7% Nitrospirae, 7.5% representatives of the TM7 candidate branch. The following representatives were also found: Acidobacteria 1.9%, Planctomycetes 1.8%, Betaproteobacteria 1.2%, Cyanobacteria 0.2% and Deltaproteobacteria 0.1%.

In the microbial community of Sample 1 (Fig. 2), sequences of Acidobacteria 17.7%, Actinobacteria (Acidimicrobiales) 13.3%, Alphaproteobacteria 6.6%, Betaproteobacteria 3.9%, and Gammaproteobacteria 9.9% were found. Almost half of the sequences have not been classified.

## 2 Experimental Design, Materials and Methods

The NOM samples were taken in sterile 50ml Falcon tubes and were stored in alcohol at −70 °C. To isolate total DNA, 0.3 g of the sample and a Genomic DNA from soil NucleoSpin® Soil kit were used. The procedure was carried out in accordance with the manufacturer's protocol.

The target fragments of 16S rRNA genes (region B3-B4) were obtained using the degenerate primers U343F (5′-CCTACGGGRSGCAGCAG-3′) and U806R (5′-GGACTACNVGGGTWTCTAAT-3′), which previously demonstrated the ability to amplify a wide range of microorganisms [2]. To obtain a library of gene fragments with the lowest number shift, we used Fusion Polymerase Q5 from New England Biolabs and low temperature annealing of the primers. The first amplification regime: 96 °C - 2′; 25*(96 °C-8″; 54 °C-20″; 68 °C-30″). The product of the first amplification was purified and used for the second PCR reaction. The second amplification regime: 96 °C - 2′; 5*(96 °C-8″; 54 °C-20″; 68 °C-30″); 20*(96 °C-8″; 60 °C-20″; 68 °C-30″). During the second PCR reaction, Illumina sequencing adapters and dual-index barcodes were attached to the target amplicon. Sequencing of the libraries was performed on Illumina MiSeq using the TG MiSeq Reagent Kit v3 (600 cycles) at the Genomics Laboratory of the IMKB SB RAS. Sequenced reads were automatically separated by barcodes.

Bioinformatic analysis of paired reads of the 16S rRNA gene was performed on the QIIME2 v.2020.2 platform [3]. Using the DADA2 tool, noise was removed, paired reads were integrated, and OTUs were built. Taxonomic classification of the obtained OTUs was carried out using the scikit-learn classifier, which was trained on fragments of 16S rRNA from the Greengenes v.13_8 database, limited by the used primers.

## Ethics Statement

The work did not involve the use of human subjects, animals, cell lines and endangered species of wild fauna and flora.

## CRediT Author Statement

**Anton V. Korzhuk:** Writing - Original Draft, Visualization; **Irina N. Myagkaya:** Project administration, Investigation; **Alexei S. Rozanov:** Visualization, Methodology; **Nikita I. Ershov:** Data Curation; **Bagai-ool Yu. Saryg-ool:** Investigation; **Victor I. Malov:** Investigation; **Maria A. Gustaytis:** Investigation; **Aleksandra A. Shipova:** Investigation; **Elena V. Lazareva:** Conceptualization; **Sergey E. Peltek:** Supervision.

## Declaration of Competing Interest

The authors declare that they have no known competing financial interests or personal relationships which have, or could be perceived to have, influenced the work reported in this article.
